# What is the role of sentinel lymph node biopsy in the management of oral cancer in 2020?

**DOI:** 10.1007/s00405-020-06538-y

**Published:** 2020-12-28

**Authors:** Remco de Bree, Bart de Keizer, Francisco J. Civantos, Robert P. Takes, Juan P. Rodrigo, Juan C. Hernandez-Prera, Gyorgy B. Halmos, Alessandra Rinaldo, Alfio Ferlito

**Affiliations:** 1 Department of Head and Neck Surgical Oncology, University Medical Center Utrecht, P.O. Box 85500, 3508 GA Utrecht, The Netherlands; 2 Department of Radiology and Nuclear Medicine, University Medical Center Utrecht, Utrecht, The Netherlands; 3 Department of Otolaryngology, Sylvester Cancer Center, University of Miami, Florida, USA; 4 Department of Otolaryngology/Head and Neck Surgery, Radboud University Medical Center, Nijmegen, The Netherlands; 5 Department of Otolaryngology, Hospital Universitario Central de Asturias-University of Oviedo, ISPA, IUOPA, Oviedo, Spain; 6 Department of Pathology, Moffitt Cancer Center, Tampa, FL USA; 7 Department of Otolaryngology/Head and Neck Surgery, University Medical Center Groningen, Groningen, The Netherlands; 8 Department of Otolaryngology, University of Udine School of Medicine, Udine, Italy; 9 International Head and Neck Scientific Group, Padua, Italy

**Keywords:** Sentinel node biopsy, Oral cancer, Lymph node metastasis, Staging, Neck dissection

## Abstract

Approximately 70–80% of patients with cT1-2N0 oral squamous cell carcinoma (OSCC) ultimately prove to have no cancer in the cervical lymphatics on final pathology after selective neck dissection. As a result, sentinel lymph node biopsy (SLNB) has been adopted during the last decade as a diagnostic staging method to intelligently identify patients who would benefit from formal selective lymphadenectomy or neck irradiation. While not yet universally accepted, SLNB is now incorporated in many national guidelines. SLNB offers a less invasive alternative to elective neck dissection (END), and has some advantages and disadvantages. SLNB can assess the individual drainage pattern and, with step serial sectioning and immunohistochemistry (IHC), can enable the accurate detection of micrometastases and isolated tumor cells (ITCs). Staging of the neck is improved relative to END with routine histopathological examination. The improvements in staging are particularly notable for the contralateral neck and the pretreated neck. However, for floor of mouth (FOM) tumors, occult metastases are frequently missed by SLNB due to the proximity of activity from the primary site to the lymphatics (the shine through phenomenon). For FOM cancers, it is advised to perform either elective neck dissection or superselective neck dissection of the preglandular triangle of level I. New tracers and techniques under development may improve the diagnostic accuracy of SLNB for early-stage OSCC, particularly for FOM tumors. Treatment of the neck (either neck dissection or radiotherapy), although limited to levels I–IV, remains mandatory for any positive category of metastasis (macrometastasis, micrometastasis, or ITCs). Recently, the updated EANM practical guidelines for SLN localization in OSCC and the surgical consensus guidelines on SLNB in patients with OSCC were published. In this review, the current evidence and results of SLNB in early OSCC are presented.

## Introduction

Cervical lymph node metastasis is the single most important prognostic factor in oral squamous cell carcinoma (OSCC), and accurate detection of cervical lymph node metastases is critical for surgical and adjuvant therapy planning. Palpation has a low sensitivity in detecting lymph node metastases and proved to be inferior compared to conventional imaging techniques, such as ultrasonography (US), computerized tomography (CT) and magnetic resonance imaging (MRI) and Fluorine-18-fluorodeoxyglucose positron emission tomography (FDG PET). US, CT and MRI imaging modalities focus on morphologic (size and homogeneity) aspects of lymph nodes and FDG PET focusses on glucose consumption. None of these imaging techniques is able to detect small or microscopic subclinical metastases and to subsequently change treatment strategies.

US-guided fine-needle aspiration cytology (USgFNAC) shows a higher sensitivity and higher specificity than CT, MRI and FDG PET, but it also has its limitations. USgFNAC is strongly dependent on the experience and skills of the ultrasonographer and cytopathologist. Moreover, sensitivity will always be limited due to the inability to detect small metastatic deposits and to eliminate sampling error. Taking into account the limitations of palpation and imaging, patients with a clinically and radiologically negative neck (cN0) still have a substantial risk of having occult metastases in the neck [1].

How to treat patients with a substantial risk of occult metastases has been a heavily discussed topic for decades, especially in surgically treated patients who do not need an approach of the neck as part of management of the primary tumor. In early-stage OSCC (cT1-T2N0), it is usually not necessary to access the neck when the primary tumor can be resected transorally and no reconstruction is necessary. However, the incidence of occult metastases is reported to be around 30% in these cases. A debate has been ongoing for many years on how to manage patients with a clinically negative neck: should all patients be treated prophylactically with an elective neck dissection, or can the neck be left untreated with a “watchful waiting” follow-up policy [2]. SLNB provides an intermediate approach between these two options, allowing for the selection of truly positive patients for further treatment.

Sentinel lymph node biopsy (SLNB) is a diagnostic staging procedure, which is nowadays also performed in OSCC. According the sentinel node concept, the first draining lymph nodes, the sentinel lymph nodes (SLNs), are most likely to harbor metastases in case of lymphogenic tumor spread and has to be identified and extirpated. The histopathological status of the SLN reflects the status of the rest of the nodal basin, and additional treatment (surgery and/or radiotherapy) of the nodal basin should be done if metastatic tumor deposits are found in the SLN. However, if the SLN is negative neck, no treatment of the nodal basin is needed and watchful waiting of the nodal basin is justified [3].

In OSCC, the concept of SLNB has evolved during the last decades. The first studies on visualization of the cervical lymphatic vessels were reported in the 1960s. In the 1980s, mapping of lymphatic drainage from specific head and neck sites was introduced. SLNB was further developed in the 1990s and after validation in this century, the procedure is now routinely performed in early OSCC in many head and neck centers. In short, the routine SLN procedure usually consists of preoperative peritumoral injections of a technetium-99 m (^99m^Tc)-labelled nanocolloid, or other radiotracer, followed by lymphoscintigraphy using planar and single photon emission computed tomography/CT (SPECT-CT) imaging. Based on the lymphoscintigraphy, the position of the SLN is marked on the skin. Subsequently the marked SLN is surgically removed, while intraoperative detection is achieved using a portable gamma probe. Finally, meticulous histopathological examination, including step serial sectioning and immunohistochemistry, of the harvested SLN is performed [3].

In 2010, we provided an update on the role of SLNB in the management of oral cancer [4]. Now, 10 years later, we want to update the knowledge on this topic by discussing the recent developments and the current role of SNLB in early OSCC.

## Sentinel lymph node biopsy as alternative for elective neck dissection

Since 2010, large single-center and multicenter studies in which treatment of the neck was only performed after positive SLNs showed a high sensitivity and negative predictive value. Because false positive results are unlikely to occur, specificity and positive predictive value will always be near 100%. To assess the diagnostic value of SLNB for the detection of occult lymph node metastases, follow-up of the untreated neck is a better reference standard than histopathological examination since missed lymph node metastases (false negative SNLB findings) will progress into clinically manifest ones and routine histopathological examination (not using step-serial sectioning with immunohistochemistry) of an END (irrespective of the SLNB result) may miss micrometastases (defined as tumor clusters > 0.2 mm and < 2 mm in size) and isolated tumor cells (ITC; defined as small clusters of cells not larger than 0.2 mm, or single cells, or fewer than 200 cells in a single cross section) up to 15% [5]. Furthermore, in analyzing the accuracy of SLNB, it is important to realize that incidence of occult lymph node metastases and follow-up time (as reference standard) affect the negative predictive value (NPV) and sensitivity, respectively. The lower the incidence, the more likely the NPV is higher. The shorter the follow-up time, the lower the incidence of delayed (missed) metastases (recurrences) and the higher the sensitivity may be.

### Single-center studies

Considering that histopathology of SLN-positive patients in combination with long-term follow-up of SLN-negative patients is the best available reference standard to define the accuracy of the SLNB procedure, we will discuss studies which used this reference standard (Table 1). Broglie et al. performed a prospective consecutive cohort analysis of 111 cT1-2N0 OSCC patients with an incidence of 40% occult lymph node metastases and found a sensitivity of 93% and NPV of 96% after a median follow-up of 37 months. Lymph node mapping consisted of preoperative dynamic and static lymphoscintigraphy and additional SPECT-CT in 34% of cases. They demonstrated that even small tumor deposits (ITC and micrometastasis), only detectable by the extensive histopathologic workup of the SLNB protocol, have a significant impact on tumor control and survival [6]. Den Toom et al. reported on the results of SLNB in 90 cT1-2N0 OSCC patients: sensitivity and NPV were 93% and 97%, respectively. The median follow-up was 18 months. In 65% of patients, SPECT/CT was performed preoperatively and additionally in all patients intraoperatively patent blue dye was peritumorally injected for visualization (coloring) of SLNs [7]. In the largest single center study of 253 cT1-2N0 OSCC patients of whom 30% had lymph node metastases Pedersen et al. using a median follow-up of 32 months as reference standard found for SNLB a sensitivity and NPV of 88%, and 95%, respectively. SPECT-CT was used in all patients. In their series, the group of Copenhagen did not yet use near-infrared (NIR) fluorescence imaging to improve the SNB procedure by facilitating intraoperative visual identification of the SLN [8]. Moya-Plana et al. reported on SNLB in 179 cT1-2N0 OSCC patients with a long term (median 5.3 years) follow-up who underwent neck dissection only if a SLN was positive and reported a sensitivity of 80% and NPV of 95%. This lower sensitivity may be due to the high number (40%) of floor of mouth tumors (see below) and long follow-up time. SPECT-CT was not used in all patients. Frozen section of SLN was used intraoperatively to perform a completion (therapeutic) neck dissection in the same session in case of a positive SLN [9]. Figures about the sensitivity of frozen sections to detect occult metastasis in SLNs were not reported. Due to tissue processing limitations and artifacts, frozen section analysis of SLNs can underestimate the true incidence of metastasis, particularly in the identification of ITC and micrometastasis. False negative SLNs may contain tiny deposits of microscopic metastatic disease, which may be missed in the examined histologic slides. Vorburger et al. compared the detection rate of occult metastases by monoslice frozen section with the definitive histopathologic work up. They found a sensitivity of frozen section of 47% which was dependent on the metastases size: for ITC 8%, for micrometastases 43%, and for macrometastases 93% [10]. Mølstrøm et al. analyzed SLNB in 220 cT1-2N0 OSCC patients with an incidence of occult metastasis of 30% and found a sensitivity of 83.3% and a NPV of 93.3% after a median follow-up of 30 months. SPECT-CT was used in the majority of patients. The authors focused on the topographical distribution of sentinel nodes and metastases (see below) [11]. Boeve et al. found a sensitivity of 85% and NPV of 94% for SLNB in detecting occult metastases (incidence 33%) in 91 cT12N0 OSCC patients after a median follow-up of 32 months [12].

**Table 1:** Results of largest studies on SLNB in OSCC patients with adequate reference standard

Study	Number	Incidence (%)	Follow-up*	Sensitivity (%)	NPV (%)	FOM tumors (%)
Single center studies						
Broglie et al. [6]	111	40	37 (2–83)	93	96	26
Den Toom et al. [7]	90	33	18 (2–62)	93	97	26
Pedersen et al. [8]	253	27	32 (1–92)	88	95	47
Moya-Plana et al. [9]	179	27	62	80	95	40
Mølstrøm et al. [11]	220	30	30 (3–111)	83	93	31
Boeve et al. [12]	91	33	32 (21–47)	85	94	30
Multicenter studies						
Flach et al. [13]	62	40	53 (5–77)	80	88	36
Schilling et al. [14]	415	26	36	86	94	25
Den Toom et al. [19]	488	22	26 (0–116)	81	93	27
Meta-analyses						
Liu et al. [16]	3566			87	94	
Yang et al. [17]	1084			92	96	0

Incidence of lymph node metastasis

*NPV* negative predictive value, *FOM* floor of mouth; percentages of included OSCC patients

*Median (range) follow-up in months

### Multicenter studies

Since 2010 also, some multicenter studies have been published with slightly lower sensitivity and specificity as can be expected. Flach et al. reported on a Dutch multicenter study in 62 cT1-2N0 OSCC patients a sensitivity was 80% and NPV 88%. SLNB was able to reduce the risk of occult lymph node metastases in OSCC patients from 40 to 8%, and enabled an excellent neck control (97.4%) [13]. In the largest multicenter study of 415 cT12N0 OSCC patients, a sensitivity of 86% and NPV of 95% were found for SNLB to detect occult lymph node metastasis (incidence 26%) after 3-year follow-up [14]. In the latter European SENT trial, all participating centers had to complete at least ten successful training SNLB procedures (validated against neck dissection) prior to recruiting to SENT. In the Dutch SNUS trial, this learning curve was not a criterion for unit participation, which is likely the reason for the lower accuracy. The importance of the learning curve was already shown in the ACOSOG Z0360 study in which the NPV of procedures performed by surgeons who entered the trial with more experience in the use of SLNB for oral cancer was higher than for less experienced surgeons [15].

### Meta-analysis

In the most recent meta-analyses, also a high sensitivity and NPV for SNLB to detect occult lymph node metastases in OSCC were found. In 1 meta-analysis comprising 66 studies (3566 OSCC patients) a pooled sensitivity of 87% and a pooled negative predictive value of 94% were found [16]. In another meta-analysis, 35 studies (with 1084 tongue squamous cell carcinoma patients) were included. The pooled overall sensitivity and NPV of SLNB were 92% and 96%, respectively [17]. In these meta-analyses, studies which used routine histopathological examination of elective neck dissection specimens as a reference standard, were included, which may result in an overestimation of the accuracy because of possible false negative histopathological results [5].

### Comparison sentinel node biopsy and elective neck dissection

Only a few studies have compared recurrence rates and survival after SLNB and END. Moya-Plana et al. compared patients who underwent systematic END (*n* = 50) to patients who underwent only neck dissection if SLNB was positive (*n* = 179). Nodal recurrence was reported in 10.0% and 7.8%, respectively. They found no statistically significant difference between the systematic ND group and the SNB group for 5-year recurrence free survival (77.7% vs 80.7%; *p* = 0.84) and for overall survival (78.7% and 76.4%; *p* = 0.73) [9]. In a recent retrospective cohort study of 8328 patients with cT1-2cN0 OCSCC who underwent staging of the neck by SLNB (*n* = 240; 2.9%) or END in the United States National Cancer Data Base from 2012 to 2015, with a median follow-up of 35.4 months, the overall 3-year survival was significantly better after SLNB (82.0%) than after END (77.5%, *p* = 0.40). However, after adjustment, overall survival was equivalent between patients who underwent SLNB versus END (adjusted hazard ratio 1.03, confidence interval 0.67–1.59). Moreover, SLNB was associated with reduced perioperative morbidity, with median length of hospital stay of 1.0 days versus 3.0 days after END (*p* < 0.001). Perioperative 30-day mortality was 0% after SLNB versus 0.7% after a recent retrospective, multicenter cohort study included 390 patients staged by END and 488 by SLNB. The overall sensitivity (84% vs. 81%) and NPV (both 93%) were comparable between END and SLNB patients. The END cohort contained more pT2 tumors (51%) compared to the SLNB cohort (23%) (*p* < 0.001). No differences were found for sensitivity and NPV between SLNB and END stratified for pT stages. In floor of mouth (FOM) tumors, SLNB had a lower sensitivity (63% vs. 92%, *p* = 0.006) and NPV (90% vs. 97%, *p* = 0.057) compared to END. Higher disease-specific survival (DSS) rates were found for pT1 SLNB patients compared to pT1 END patients (96% vs. 90%, *p* = 0.048) [19]. In the French Senti-MER trial, the only randomized clinical trial, 307 cT1-2N0 were randomized between END and SNLB. After a mean follow-up of almost 5 years, the 2- and 5-year neck node recurrence free survival for END and SLNB were comparable: 89.6% vs. 90.7% and 89.6% vs. 89.4%, respectively. Also, locoregional free, disease-specific and overall survival were not different for both groups. Lower morbidity was observed in the SLNB arm during the first year post-surgery [20]. See also Table 1.

Several studies reported the differences in complication rates, postoperative morbidity and cost-effectiveness in favor of the SLNB compared to the END procedures [9, 21–27]. Moya-Plana et al. found a significant higher complication rate for patients after neck dissection as compared to SNB only patients: 28% vs. 8% (*p* < 0.0001) presented at least one complication [9]. Using different scoring systems, Murer et al. found a higher shoulder morbidity for END as compared to SNLB [23]. Govers et al. performed a modeling study of the cost-effectiveness and found SLNB to be the most cost effective strategy for diagnosing and treating OSCC patients [29].

Due to the overall predominantly good performance, SLNB was quickly adopted during the last decade as diagnostic staging method and is now incorporated in many national guidelines including those from The Netherlands, United Kingdom (NICE) and USA (NCCN). Compared to the routinely performed END, its minimally invasive design combined with a high sensitivity for detecting occult metastases offers the possibility to reduce the number of surgically overtreated patients. Although SLNB for cT1-2N0 OSCC is also associated with reduced morbidity, costs and length of hospital stay, increased quality of life and equivalent overall survival compared with END it remains rarely used in the United States [18]. Contrary, in Europe, SLNB is increasingly used in cT1-2N0 OSCC patients. A survey in the Netherlands revealed that in 2006 none of the head and neck centers used SLNB routinely [28], while in 2014, four centers [29] and nowadays, seven of the eight major centers of the Dutch Head and Neck Society (NWHHT) use SLNB routinely in cT1-2N0 OSCC patients.

## Improved staging by sentinel lymph node biopsy

The SLNB procedure can assess the individual drainage pattern. In addition, despite some variability among different studies, most histological protocols for the evaluation of SLNB typically involves consist of sectioning of the lymph node at 2 mm thickness, subsequent multiple levels and the use immunohistochemistry for cytokeratin. This approach allows for the identification of isolated tumor cells and micrometastases. Such histological protocols would be impractical for a large specimen derived from an END. In this way, one could argue that SLNB provides better results in the detection of lymph node metastasis and therefore improved staging as compared to END with routine histopathological examination.

Several studies showed a significant difference between SLNB-positive and SLNB-negative patients. Broglie et al. found an overall survival (OS), disease specific survival (DSS) and disease-free survival (DFS) at 3 years for SLNB-negative and SLNB-positive patients of 98% and 71%, 95% and 76%, and 98% and 73%, respectively [6]. In a study of Den Toom et al. OS and DFS for SLNB negative were 100% and 84% compared to 73% and 88% for SLNB-positive patients, respectively [7]. This significant difference for OS was also found in the SENT trial [14].

Broglie et al. were the first to demonstrate that even small tumor deposits only detectable by the extensive histopathologic workup of the SLNB protocol have a significant impact on tumor control and survival in early OSCC. Forty-nine of 111 patients (38%) had positive SLNs, 10 had ITCs, 19 had micrometastases, and 13 had macrometastases. They found a statistically significant difference between the SLN-negative group and ITCs, micrometastases and macrometastases groups in different survival estimates [7]. Pedersen et al. found also a shorter DFS for patients with ITC or micrometastases compared patients without metastases [8]. In the SENT trial a significant difference was found for OS between ITC, micrometastasis and macrometastasis [14]. These data underline the clinical importance of detecting ITCs and micrometastasis in OSCC.

This means that the presence of positive SLNs and size of tumor deposits within these SLNs, i.e. ITCs, micrometastases and macrometastases, may be used to predict survival and individualized treatment planning. As it is not feasible to investigate all lymph nodes in an END specimen by step serial sectioning and immunohistochemistry, SLNB will more accurately stage the neck enabling better prognostication and more individualize additional treatment.

On the other hand, free soft tissue disease (defined as a metastatic carcinoma in the soft tissue of the neck with no evidence of a lymph node architecture) can probably not be detected by SLNB [30].

### Contralateral neck

SLNB stages both the ipsilateral as well as the contralateral neck in lateralized cT1-2N0 OSCC patients, whereas the contralateral neck is usually not addressed by END in cT1-2N0 OSCC not involving the midline. Although the reported incidence of contralateral lymph node metastases in lateralized early-stage OSCC is relatively low, underdiagnosis of the contralateral clinically negative neck is undesirable, especially since the presence of contralateral lymph node metastases from OSCC have been associated with poor DSS [31]. Contralateral lymphatic drainage and positive SLNs have been reported in several studies. Moya-Plana et al. found among 195 unilateral primary tumors contralateral SLNs in 12.8% [9]. In the Dutch SNUS trial, contralateral drainage was observed in 13% of well-lateralized tumors [13]. In the European SENT trial, unilateral drainage only was found in 40% of midline OSCC and contralateral drainage in 12.4% of well-lateralized OSCC [14]. Mølstrøm et al. investigated the topographical distribution of SLNs and metastases from cT1-T2N0 OSCC. In 28.5% of patients with midline tumors, only unilateral lymphatic drainage on lymphoscintigraphy was observed. Patients with lateralized OSCC had unexpected bilateral or contralateral drainage patterns in 22.6% and contralateral positive SLNs in 9%, which would have been missed by elective neck dissection of the ipsilateral neck [11].

### Previously treated neck

Patients with a history of surgery or radiotherapy in the neck may have aberrant lymphatic drainage caused by disruption of lymphatic channels. Therefore, elective treatment of the same levels at risk as in the primary setting may not be appropriate. SNB in the pretreated neck appears to be feasible and renders an assessment of the individual lymphatic drainage pattern, compensating for a potential variability after previous treatment of the neck. Unexpected lymphatic drainage patterns (first SLN at level IV or V and contralateral neck in well-lateralized tumors) were found in 30–67% of cT1-2N0 OSCC patients with a previously treated neck. SNLB was able to upstage the neck of these patients in 7% [32, 33].

Therefore, SLNB can be used to assess individual and unexpected lymphatic drainage patterns allowing for tailoring treatment of the neck reducing under- and overtreatment, also in a pretreated neck.

## Selection of patients for sentinel lymph node biopsy

Traditionally SLNB in OSCC patients is reserved for tumors which can be transorally resected without opening the neck for resection of the primary tumor or reconstruction and a clinically negative neck. In the 7th edition of the TNM classification these tumors were generally staged cT1-2N0; however, in the 8th edition, the depth of invasion is also taken into consideration. This results in upstaging T2 tumors with deep invasion (> 10 mm) to T3 tumors, which may be still suitable for resection via transoral approach.

### Depth of invasion

Some institutes use depth of invasion for the decision to perform an END or watchful waiting and continued this for a decision to perform SLNB or END. Although depth of invasion (DOI) is associated with risk of lymph node metastases and survival a cut-off DOI for SLNB or END is difficult to determine. Den Toom et al. investigated if extent of DOI can predict occult nodal disease in 199 patients with cT1-2N0 (7th TNM) OSCC staged by SNLB. The mean DOI of patients with and without lymph node metastasis was significantly different: 6.6 mm vs. 4.7 mm (*p* = 0.003). The ROC showed an area under the curve of 0.65 with a most optimal cut-off point of 3.4 mm DOI (sensitivity 83% and specificity 47%). However, regional metastases were still found in 15% of patients with DOI ≤ 3.4 mm and it was concluded that DOI seems to be an insufficient predictor to assess which cT1-2N0 OSCC patients can refrain from SLNB. Therefore, it was suggested that staging of the neck using SLNB in early-stage oral cancer patients should also be performed in tumors with limited DOI and probably in T3 OSCC ≤ 4 cm diameter (thus tumors upstaged from T1-2 to T3 by 8th TNM) [34]. An exception is minimally invasive lesions with less than one mm depth of invasion, where lymphatic metastases are exceedingly rare. These are lesions that are often initially diagnosed as carcinoma in situ, and later minimal invasion is found on the resection [35].

### Floor of mouth

Generally, SLNB has proven to reliably stage the clinically negative neck in early-stage OSCC with high sensitivity and negative predictive value. However, in floor of mouth OSCC, the accuracy to detect occult lymph node metastasis is significantly lower. In a series of 488 cT1-2N0 OSCC patients, SLNB had a lower sensitivity in FOM tumors than in non-FOM locations: 63% and 86% (*p* = 0.008), respectively [19].

When SNLs are located in close vicinity of the tracer injection site, due to the limited resolution of the γ-camera and SPECT, the hotspot of the tracer injection site can hide adjacent SLNs. This ‘shine-through phenomenon’ hampers discrimination between tracer injection site and SLNs. A lower accuracy for SLNB can result in missing occult lymph node metastasis, which will inevitably develop into clinical manifestation of disease and consequently induce a poor oncological prognosis.

There are some indications that this shine through phenomenon explains the lower sensitivity of SLNB in FOM OSCC in the literature. In the series of Pedersen et al., six of the nine false negative SLNB procedures were in FOM tumors and six of these nine regional recurrences were upstream from the levels initially explored (four in level IA; two in level IB) [8]. In the study of Moya-Plana et al., 6 of the 46 (13%) patients with a positive SLNB, SLNs were positive in II–III, and positive lymph nodes were found in IB area upon completion neck dissection [9]. In the study of Den Toom et al. in 7 of the 11 (64%), FOM patients with a false negative SLNB developed a regional recurrence in level I [7].

These findings suggest that in FOM OSCC occult metastasis are frequently missed by SLNB due to the shine through phenomenon and, therefore, the use of the standard SLNB technique in FOM OSCC is debatable. To overcome this limitation of SLNB in FOM OSCC, a superselective neck dissection of the preglandular triangle of level I was described by Stoeckli et al. and is now routinely used in some centers. They evaluated this technique in 40 consecutive and prospectively enrolled FOM OSCC patients. Eleven of 22 (50%) SLNS were only detected intraoperatively. Using this technique in addition to the standard technique a false negative rate of 8.3% and negative predictive value of 96.4% were reported [36]. Therefore, it is advised not to perform SLNB routinely or do a superselective neck dissection of the preglandular triangle of level I in FOM OSCC until the SNLB procedure is improved.

## Techniques for improving sentinel lymph node biopsy accuracy

Technical developments are needed to bring the diagnostic accuracy of SLNB for early-stage OSCC to a higher level, particularly for FOM tumors. Some techniques which improve SNLB are already implemented in current routine SLNB, while other techniques are still under investigation.

### Single photon emission computed tomography with computed tomography

Historically, visualization of SLNs is routinely carried out with static and dynamic planar lymphoscintigraphy. SPECT/CT was already introduced in oral cancer in 2003 [37]. Although most studies concluded that SPECT/CT improves localization of the SLNs and detection of additional SLNs [38], it took some years to become part of the standard lymphoscintigraphy protocol [39]. SPECT-CT has especially advantages for tumors with close proximity to the SLN and regions with complex lymphatic drainage which is the case in OSCC, particularly FOM tumors [40]. Den Toom et al. investigated the additional value of SPECT/CT to planar images for the identification SLNs in 66 patients with early-stage OSCC. SPECT-CT identified 15 additional SLNs in 14 patients (22%). In 13% of these additional SLNs, the only metastasis was found, resulting in an upstaging rate of 3%. In 20% of the patients with at least one positive SLN, the only positive SLN was detected by the addition of SPECT/CT. SPECT/CT was considered to add important anatomical information in two patients (3%) [41]. The addition of SPECT/CT to planar lymphoscintigraphy is now recommended for the identification of more (positive) SLNs and better topographical orientation for surgery in SLNB for early-stage OSCC [42].

### Fluorescence imaging

For intraoperative visualization of SLNs, traditionally peritumoral injection of blue dye has been traditionally used. Because of its very limited additional value [43], the use of blue dye in SLNB was abandoned by many. Near-infrared (NIR) fluorescence imaging is nowadays increasingly used in SLNB. NIR dyes have the advantage to exhibit reasonable tissue penetration of excited and emitted light with negligible auto-fluorescence, resulting in high target-to-background contrast. NIR fluorescence imaging provides high-resolution images which can be obtained in real time during the surgical procedure, even if the structure of interest is covered by some tissue (in contrast to blue dye). Another advantage of NIR fluorescence imaging is that it is much better suited for detection of SLNs close to the primary, because there is negligible influence of fluorescence signal coming from the injection site given the limited penetrance of the emitted light signal through surrounding tissue. Because of its easy availability indocyanin green (ICG) is the most frequently used NIR-fluorescent compound. Because ICG alone has a poor retention in SLN it is combined with nanocolloidal albumin. Van den Berg et al. were the first to evaluate the added value of intraoperative fluorescence imaging to the conventional radioguided procedure in 14 OSCC patients using ICG-^99m^Tc-nanocolloid, a hybrid tracer that is both radioactive and fluorescent. SLNs were detected preoperatively by lymphoscintigraphy, including SPECT-CT, and intraoperatively with a gamma-probe and NIR camera. In four patients, a SLN located close to the primary injection site could only be intraoperatively localized using fluorescence imaging. In one patient this SLN contained a metastasis. In 4 of the 14 OSCC patients where the SLN was located close to the primary injection site the SLN could only be localized by fluorescence imaging [44]. In a study of Christensen et al. in 30 OSCC patients using the hybrid tracer ICG-^99m^Tc-nanocolloid SLNB revealed a total of 94 SLNs of which 11 (12%) could only be identified by NIR. The majority of those were located in level I close to the primary tumor [45]. The combination of ICG and a radiopharmaceutical enables the identification of SLNs more easily and rapidly than using a radiopharmaceutical alone. Intraoperative fluorescence guidance seems of particular value when SLNs are located in close proximity to the injection site. Other tracers with improved optical properties have been tested in preclinical settings [46, 47].

### Novel tracers

Radiolabelled tracers other than colloidal albumin with other characteristics may improve intraoperative differentiation between SLN and injection site [48]. A new radioactive agent, ^99m^Tc-tilmanocept (Lymphoseek ®, Navidea Biopharmaceuticals, Inc.), has been specifically designed for SLN identification and is registered for this purpose in both the USA and Europe. ^99m^Tc-tilmanocept is a small sized receptor (CD206) targeted SLN detection agent. Due to its proposed rapid clearance from the injection site, rapid uptake and high retention within the SLN, as well as low uptake by the remaining (higher echelon) lymph nodes, ^99m^Tc-tilmanocept may particularly be of benefit in floor of mouth tumors and other head and neck tumors with complex drainage patterns and close spatial relation to the SLNs [49].

A multicenter validation study using ^99m^Tc-tilmanocept for SLNB in head and neck squamous cell carcinoma of skin and (mainly) oral cavity showed an SLN identification rate of 97.6%, a false negative rate of 2.56% and an NPV of 97.8%. Of note, these favorable outcomes were also obtained in FOM cancers, which strengthened the idea that ^99m^Tc-tilmanocept may diminish the shine through effect and improve the SLN detection rate for this subsite [50].

A recent prospective within-patient evaluation study compared ^99mTc^Tc-tilmanocept with ^99m^Tc-nanocolloid for SLN detection in 20 patients with early-stage OSCC, who underwent lymphoscintigraphy with both tracers. ^99m^Tc-tilmanocept had a higher injection site clearance, but at the same time, a lower uptake in the SLN, resulting in a comparable SLN to injection site ratio, using ^99m^Tc-nanocolloid [51]. ^99m^Tc-tilmanocept is registered at the FDA and EMA using an activity of 74 MBq, which is lower than activities routinely used with ^99m^Tc-nanocollidal tracers. The low radioactivity used results in relatively low uptake in SLNs of ^99m^Tc-tilmanocept which may limit intraoperative detection of SLNs. This might be overcome by a higher injection activity. Larger trials, preferably multicenter randomized clinical trials, are needed to determine if ^99m^Tc-tilmanocept can improve SLNB in OSCC patients.

Several other techniques are currently under development in OSCC patients. These techniques include the use of PET-CT, CT lymphography, MRI lymphography using contrast agents or superparamagnetic iron oxide, contrast-enhanced ultrasound mapping using microbubbles, and freehand SPECT [52].

## Subsequent treatment after positive sentinel lymph node biopsy

Because there is no reliable method for detection or prediction of non-SLN metastasis, patients with positive SLNB undergo a subsequent (completion) neck treatment. In an analysis of the reported data in the literature, a relation between the size of tumor deposits in the SLN and the risk of a non-SLN metastasis in the completion neck dissection was found: 13% for ITC, 20% for micrometastasis and 40% for macrometastasis [53]. Since in patients with ITC in a SLN has a substantial probability of non-SLN metastasis in the neck, treatment of the neck remains mandatory after any category of positive SLNB in early-stage OSCC patients.

### Subsequent neck dissection versus radiotherapy

Both neck dissection and radiotherapy can treat eventual non-SLN lymph node metastasis well. The choice of definitive treatment modality of the neck after positive SLNB is highly dependent on the treatment modality needed for adjuvant treatment of the primary tumor and eventual previous treatment of the neck.

In a multicenter retrospective study of 107 OSCC patients with positive SLNB ITCs were detected as the largest metastatic deposit in SLNB in 15 patients (14%), micrometastasis was detected in 31 patients (29%) and macrometastasis in 61 patients (57%). A positive SLNB was followed by an additional (selective) neck dissection in 86% of the patients). Ten patients received radiotherapy on the neck and remained free of regional recurrence. Five patients refused any additional treatment [19]. In a study of Pedersen et al., the overall neck control rate in early-stage OSCC patients who underwent SLNB was 96% (243 of 253 patients). The regional recurrence rates for the 68 SLNB-positive patients who underwent subsequent neck dissection (n = 36 of whom 7 with adjuvant radiotherapy), patients who received radiotherapy only (n = 19) and patients were received no additional treatment and subsequently underwent close clinical follow-up (n = 13) were 11%, 16% and 31%, respectively [8].

From these studies, it may be concluded that the first choice for the treatment of the neck after positive SLNB is neck dissection; however, definitive radiotherapy is probably a reliable alternative.

### Extent of neck disssection

A report of the European multicenter SENT study on 109 OSCC patients with positive SLNB showed additional (non-SLN) metastases in 34.4% of the neck dissection specimens. The risk of non-SLN metastases outside the adjacent basins of the positive SLN was low (7.1%) [54]. In a report on the 3-year results of the SENT study 94 patients with a positive SLNB who underwent subsequent neck dissection were analyzed. In 85% of cases, no further positive nodes were found in the completion specimen. Of the 15 patients with additional positive non-SLN, 13 (87%) were located in the same neck level as the SLN or an adjacent neck level [14]. In another study of 36 OSCC patients with positive SLNB, all non-SLN metastases were found in levels I–IV except for one in level V. In this latter patient, two positive SLNs and five additional non-SLNs metastases were found. In four of the six (67%) patients with non-SLN metastasis, these were only found in nonadjacent levels [53]. Pedersen et al. analyzed 36 OSCC patients with positive SNLB who underwent subsequent neck dissection. Additional lymph node macrometastases were histopathologically identified in only 2 of the 36 patients [8]. In a study on the topographical distribution of SLNs and non-SLN metastases in 220 patients with early-stage OSCC and lymph node metastases, 53 patients had positive SLNB and underwent subsequent neck dissection. Metastatic involvement of neck level IV was rare and only observed in two patients with anterior tongue cancer. No patients had level V involvement [11].

The results of these studies support the use of a selective neck dissection (levels I–III/IV), in cases of a SLNB-positive neck in whom additional neck dissection is indicated. If future larger studies report more specifically on the level involved by non-SLN metastases more tailored (super)selective neck dissections may be defined.

## Guidelines

In 2009, joint practice guidelines for radionuclide lymphoscintigraphy in early-stage OSCC were published to outline the at that time best practice guidelines for SLN localization in OSCC. These guidelines were prepared by a multidisciplinary expert panel of surgeons, nuclear physicians and pathologists under the joint auspices of the European Association of Nuclear Medicine (EANM) Oncology Committee and the Sentinel European Node (SENT) Trial Committee [55]. In 2018, the eighth international symposium for sentinel node biopsy in head and neck cancer was held. This consensus conference aimed to update the multidisciplinary SLNB guidelines for nuclear medicine, surgery and pathology in early-stage OSCC. Recently, the updated EANM practical guidelines for sentinel lymph node localization in OSCC were published [42]. Also, recently surgical consensus guidelines on SLNB in patients with OSCC were reported [56]. Unfortunately, consensus was not achieved in all areas, highlighting the need for more research on SLNB in OSCC. Large registries may be helpful to further improve these guidelines in the near future.

## Limitations

This review focuses on SLNB in clinical practice of early-stage OSCC patients. In this review, only results of many studies on different topics are described. Some recent meta-analysis [16, 17] on the diagnostic accuracy of SLNB are mentioned. Moreover, the only randomized clinical trial comparing SLNB and END, which is very recently reported, is included. For other topics, data are limited and probably too heterogeneous to allow for high-quality meta-analysis. Moreover, only routinely used procedures are described, while many promising new developments, e.g. MR lymphography, CT lymphography, PET lymphoscintigraphy and contrast-enhanced lymphosonography, are under investigation to improve SLNB [57]. SLNB is not routinely used in other head and neck sites, because most of these tumors are treated non-surgically, i.e. radiotherapy with or without chemotherapy, and peri-tumoral injections are more difficult to perform than in the oral cavity, e.g. larynx [58, 59]. However, using these techniques, the SLNB procedure can also be used for individualized prophylactic neck irradiation [60, 61]. Although SLNB is a reliable technique, it is good to realize that it has also some limitations: SLNB is an invasive technique and since no intraoperative reliable technique to examine the SLNB is yet available, an eventual subsequent neck dissection has to be performed in a second-stage procedure [62].

## Conclusion

In conclusion, detection of lymph node metastases in early-stage OSCC using SLNB is a good alternative for END, except for floor of mouth tumors. SLNB potentially stages the neck more accurately enabling better prognostication and more individualized additional treatment. The main limitations of SLNB are the invasive and complex procedure and in SLNB-positive patients a subsequent neck dissection as second-stage procedure. Several techniques, e.g. MR lymphography, CT lymphography, PET lymphoscintigraphy and contrast-enhanced lymphosonography, are currently under development in OSCC patients with the aim to improve the diagnostic accuracy of SLNB for OSCC, particularly for FOM tumors, and to allow the use of SNLB for other purposes, e.g. individualized prophylactic neck irradiation. Treatment of the neck, although limited to levels I–IV, remains mandatory after any category of positive SLNB.
